# Incorporating Polygenic Risk Scores in the ACE Twin Model to Estimate A–C Covariance

**DOI:** 10.1007/s10519-020-10035-7

**Published:** 2021-02-01

**Authors:** Conor V. Dolan, Roel C. A. Huijskens, Camelia C. Minică, Michael C. Neale, Dorret I. Boomsma

**Affiliations:** 1 Netherlands Twin Register, Department of Biological Psychology, Vrije Universiteit Amsterdam, Transitorium 2B03, Van der Boechorststraat 7, 1081 BT Amsterdam, The Netherlands; 2 Virginia Institute for Psychiatric and Behavioral Genetics, Virginia Commonwealth University, 1-156, P.O. Box 980126, Richmond, VA 23298-0126 USA; 3 Stanley Center for Psychiatric Disease, Broad Institute of MIT and Harvard, Cambridge, MA 02142 USA; 4 Amsterdam Public Health Research Institute, Amsterdam Medical Centre, Amsterdam, The Netherlands; 5 Analytic and Translational Genetics Unit, Massachusetts General Hospital, Boston, MA 02114 USA

**Keywords:** Classical twin design, Polygenic risk scores, A–C covariance, Identification, Statistical power

## Abstract

The assumption in the twin model that genotypic and environmental variables are uncorrelated is primarily made to ensure parameter identification, not because researchers necessarily think that these variables are uncorrelated. Although the biasing effects of such correlations are well understood, a method to estimate these parameters in the twin model would be useful. Here we explore the possibility of relaxing this assumption by adding polygenic scores to the (univariate) twin model. We demonstrate that this extension renders the additive genetic (A)—common environmental (C) covariance (σ_AC_) identified. We study the statistical power to reject σ_AC_ = 0 in the ACE model and present the results of simulations.

**Supplementary Information:**

The online version of this article (10.1007/s10519-020-10035-7) contains supplementary material, which is available to authorized users.

## Introduction

The classical twin design (CTD; Eaves et al. 1978; Jinks and Fulker 1970) has been one of the most productive genetically informative designs in the study of human traits (Polderman et al. 2015). Twin studies have contributed greatly to our knowledge concerning genetic and environmental contributions to individual differences in psychological and medical traits, disease phenotypes and 'omics' variables (van Dongen et al. 2012). Multivariate and longitudinal extensions of the CTD have provided insights into the etiology of comorbidity and stability of traits and disorders. It is well understood that the correct interpretation of results based on the CTD depend on the tenability of the model assumptions (Eaves et al. 1977; Jinks and Fulker 1970; Plomin et al. 2016). The main assumptions of the CTD concern genotype-environment covariance (assumed to be absent), genotype-environment interaction (assumed to be absent), the equal environment assumption (environment does not cause larger resemblance in MZ than in DZ twins), and parental mating (assumed to be random, or that parental resemblance is due to social homogamy rather than phenotypic assortment). Given these assumptions, the results from the CTD can provide unbiased estimates of additive genetic (A), unshared environmental (E), and common environmental (C) and dominance (D) variance components. The effect of violations of these assumptions are well understood (Verhulst and Hatemi 2013; Purcell 2002; Keller et al. 2010), so that estimates of variance components obtained in the twin model may be interpreted in the light of possible model violations.

Many papers have been devoted to the detection and accommodation of model violations, either within the CTD (e.g., Purcell 2002; Molenaar et al. 2012; Eaves and Erkanli 2003; Carey 1986; Dolan et al. 2014; Beam and Turkheimer 2013), or in extended designs (e.g., Plomin et al. 1985; Narusyte et al. 2008; Neale and Fulker 1984; Fulker 1988; D’Onofrio et al. 2003; Keller et al. 2009; Heath et al. 1985; Maes et al. 2006). The aim of the present paper is to demonstrate that the incorporation of polygenic risk scores (PRSs) in the classical twin design allows one to estimate the covariance between A and C (σ_AC_). In the study of childhood intelligence, σ_AC_ > 0 is considered plausible, stemming from a process of cultural transmission (Keller et al. 2009; Fulker 1988), which gives rise to *passive* genotype-environment covariance in children (Plomin et al. 1977; Scarr and McCartney 1983, Knafo and Jaffee 2013; Kendler 2011; Rutter and Silberg 2002).

Measured genetic variables have been incorporated in genetically informative designs with various aims, apart from the gene finding of traditional linkage or combined linkage-association analysis (e.g., Fulker et al. 1999; Neale 2000). For instance, van den Oord and Snieder (2002) presented an extended twin model with measured genetic variables to test association in the presence of population stratification and to test causal relationships. Neale et al. (2000) partitioned variation in serum APOE levels into that associated with the APOE locus and residual genetic variance. In a study of attention problems, van Beijsterveldt et al. (2011) incorporated measured candidate gene information on SNPs in the serotonergic, dopaminergic system and the BDNF gene. The effect of SNPs was tested on a latent factor that summarized multiple assessments of attention problems across childhood. Minică et al. (2018) presented an integration of the CTD and Mendelian randomization method, in which PRSs feature as genetic instruments.

The use of measured genetic information specifically to study genotype-environment covariance is relatively new. Bates et al. (2018) and Kong et al. (2018) proposed the use of polygenic scores based on transmitted and non-transmitted alleles from parents to offspring to detect the effects of non-transmitted alleles on phenotype outcomes in their children. Warrington et al. (2018) used structural equation modeling to determine the fetal and maternal effects of measured genetic variants on birthweight, thus revealing genotype-environment covariance. Cheesman et al. (2020) applied PRSs in an adoption design to detect (passive) gene-environment correlation in educational attainment. Selzam et al. (2019) used PRSs measured in DZ twin pairs to demonstrate the presence of gene-environment correlation for cognitive abilities, and the mediating role of social economic status therein. Wertz et al. (2018) used PRSs in a parent and offspring design to demonstrate the gene-environment correlation originating in parental behavior.

The present aim is to incorporate PRSs in the twin design with the aim of estimating A–C covariance. This approach allows us to determine the presence of A–C covariance, but sheds no light on the process that gave rise to the A–C covariance. For instance, the A–C covariance may be due to active (e.g., niche picking), passive (e.g., cultural transmission) or evocative processes (Plomin et al. 1977). The outline of this paper is as follows. First, we present the classical twin model, and the model extended with PRSs. Second, given the model for PRSs in MZ and DZ twins, we address the issues of identification and statistical power. Third, we present the results of a small simulation to determine the effects of using estimated weights in calculating PRSs (i.e., the standard procedure) in comparison to exact known weights.

## The Twin Model with Polygenic Risk Scores: A–C Covariance

Let Ph denote the phenotype of interest, and let GV_k_ denote the k-th genetic variant (GV) contributing to the variance of Ph, where k = 1…K, and K is the number of GVs. We limit our presentation to diallelic GVs (e.g., SNPs) with additive effects (additively coded, e.g., 0, 1, or 2). The phenotype Ph is modeled as follows:

$${\text{Ph}} = {\text{b}}_{0} + \sum\nolimits_{k = 1}^{K} {b_{k} } {\text{GV}}_{{{\text{ki}}}} + {\text{C}}_{{\text{i}}} + {\text{E}}_{{\text{i}}} ,$$

where b_0_ is the intercept, b_k_ is the k-th regression coefficient, subscript i denotes person, and E and C represent unshared and shared environmental factor scores of individual i. In the classical twin model, under the assumptions mentioned in the introduction, the variance of Ph is decomposed, in the ACE model, into the components σ^2^_A_, σ^2^_C_, and σ^2^_E_:

$${\upsigma }^{2}_{{{\text{Ph}}}} = {\upsigma }^{2}_{{\text{A}}} + {\upsigma }^{2}_{{\text{C}}} + {\upsigma }^{2}_{{\text{E}}} .$$

The additive genetic variance equals the sum of the contributions of the individual GVs and their covariances (σ_GVk,GVl_) attributable to linkage disequilibrium:

$${\upsigma }^{2}_{{\text{A}}} = \sum\nolimits_{{{\text{k}} = 1}}^{{\text{K}}} {{\text{b}}_{{\text{k}}}^{2} {\upsigma }^{2}_{{{\text{GVk}}}} } + \sum\nolimits_{{{\text{k}} = 1}}^{{\text{K}}} {\sum\nolimits_{{{\text{l}} = 1,{\text{l}} \ne {\text{k}}}}^{{\text{K}}} {{\text{b}}_{{\text{k}}} {\text{b}}_{{\text{l}}} } } \,\,{\upsigma }_{{{\text{GVk}},{\text{GVl}}}}$$

Given σ_AC_ ≠ 0, we have (assuming no AE covariance)

$${\upsigma }^{2}_{{{\text{Ph}}}} = {\upsigma }^{2}_{{\text{A}}} + {\upsigma }^{2}_{{\text{C}}} + {\upsigma }^{2}_{{\text{E}}} + 2{\upsigma }_{{{\text{AC}}}} ,$$

where σ_AC_ is the covariance of A and C. In this model, the parameter σ_AC_ is not identified. If we assume σ_AC_ = 0, while in truth σ_AC_ > 0, the variance σ^2^_C_ is biased in the twin model, as σ_AC_ acts as C, thus inflating the estimate of σ^2^_C_ in the standard ACE model (see Purcell 2002; Verhulst and Hatemi 2013). If in truth, σ_AC_ < 0, the MZ and DZ twin correlations suggest the presence of dominance variance (D).

Given estimates of the regression coefficients (b_k_) obtained in independent genome-wide association studies (GWASs), the PRS can be calculated ${\sum }_{\mathrm{l}=1}^{\mathrm{L}}{\mathrm{b}}_{\mathrm{l}}$ GV_li_ (Purcell et al. 2009; Evans et al. 2009; Dudbridge 2013), where the set of L GVs is a subset of the K GVs. The set of L GVs may be chosen on the basis of the p value of the individual GVs or other considerations. Let the PRS equal p*A_p_, with variance p^2^*σ^2^_Ap_. The scaling parameter p accommodates the fact that the phenotype Ph and the PRS are not measured on the same scale. Let A_q_ denote the residual additive genetic variable. The model is now:

$${\text{PRS}}_{{\text{i}}} = {\text{p}}\,{\text{A}}_{{{\text{pi}}}}$$

$${\text{P}}_{{{\text{Phi}}}} = {\text{b}}_{0} + {\text{A}}_{{{\text{pi}}}} + {\text{A}}_{{{\text{qi}}}} + {\text{C}}_{{\text{i}}} + {\text{E}}_{{\text{i}}} ,$$

**Fig. 1:**
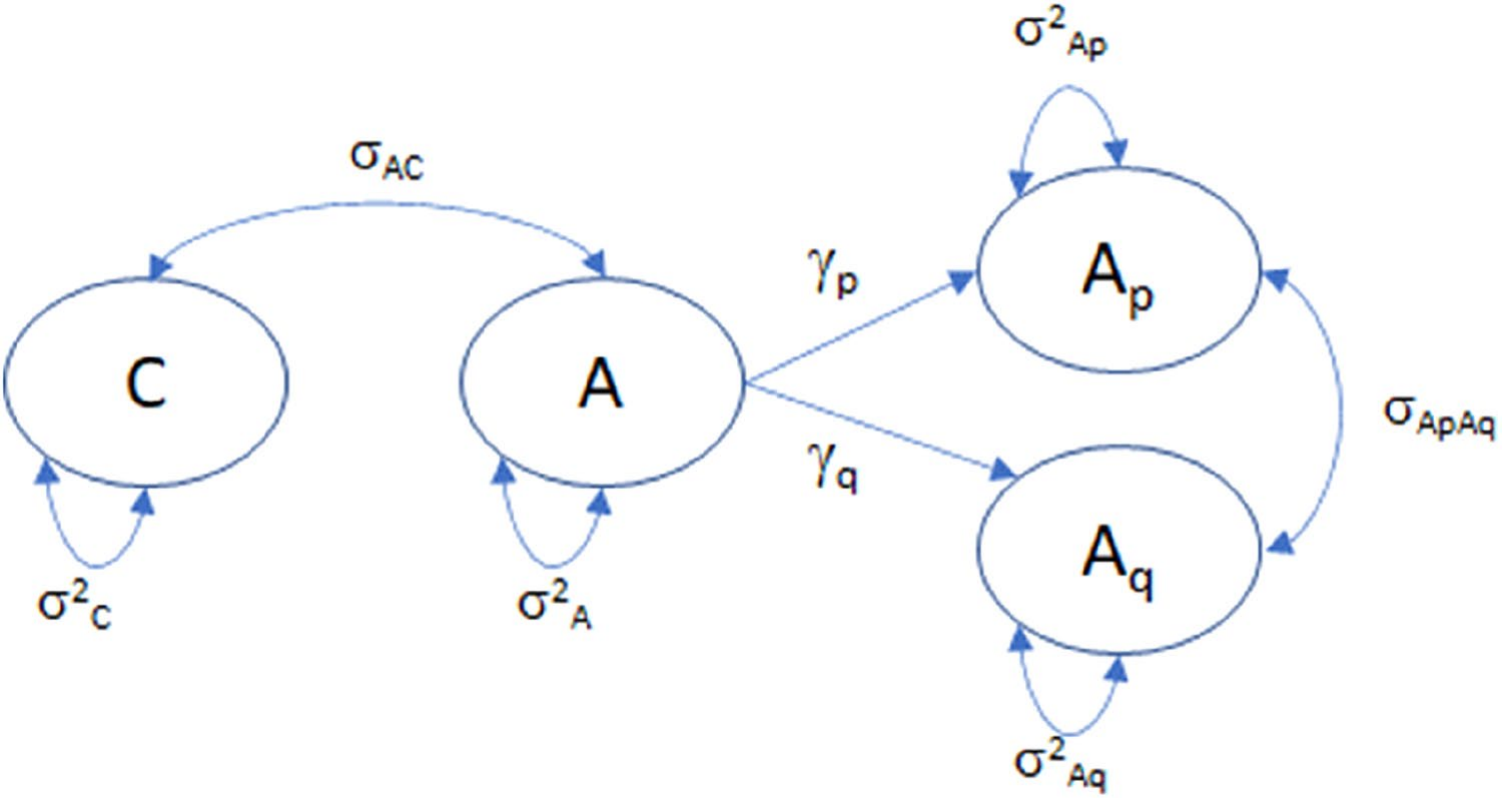
The covariance between C and A_p_ and A_q_ are derived as σ_AC_γ_p_ and σ_AC_γ_q_, respectively, where γ_p_ = σ^2^_Ap_/σ^2^_A_ and γ_q_ = σ^2^_Aq_/σ^2^_A_

The variance decomposition of the additive genetic variable A and the phenotype Ph are:

$${\upsigma }^{{2}}_{{{\text{PRS}}}} = {\text{p}}^{{2}} {\upsigma }^{{2}}_{{{\text{Ap}}}}$$

$${\upsigma }^{{2}}_{{\text{A}}} = {\upsigma }^{{2}}_{{{\text{Ap}}}} + {\upsigma }^{{2}}_{{{\text{Aq}}}} + 2{\upsigma }_{{{\text{ApAq}}}}$$

$${\upsigma }^{{2}}_{{{\text{Ph}}}} = {\upsigma }^{{2}}_{{{\text{Ap}}}} + {\upsigma }^{{2}}_{{{\text{Aq}}}} + {\upsigma }^{{2}}_{{\text{C}}} + {\upsigma }^{{2}}_{{\text{E}}} + 2{\upsigma }_{{{\text{AC}}}} ,$$

where σ_AC_ = σ_ApC_ + σ_AqC_. The parameters σ_ApC_ and σ_AqC_ are the covariances of A_p_ and C and of A_q_ and C, respectively. The parameter σ_ApAq_ is the covariance of the additive variables A_p_ and A_q_. We parameterize the covariance terms σ_ApC_ and σ_AqC_ as a function of the single covariance term σ_AC_ as follows. We derive the coefficient γ_p_ by tracing from C to A (C ↔ A with coefficient σ_AC_), and then from A to A_p_ (A → A_p_) where γ_p_ is the regression coefficient in the regression of A_p_ on A. We do the same with A_q_ using γ_q_. The path diagram is shown in Fig. 1.

We thus obtain the constraints:

$${\upsigma }_{{{\text{ApC}}}} = {\upgamma }_{{\text{p}}} {\upsigma }_{{{\text{AC}}}} ,$$

$${\upsigma }_{{{\text{AqC}}}} = {\upgamma }_{{\text{q}}} {\upsigma }_{{{\text{AC}}}} ,$$

where

1 $${\upgamma }_{{\text{p}}} = {{\left\{ {{\upsigma }^{{2}}_{{{\text{Ap}}}} + {\upsigma }_{{{\text{ApAq}}}} } \right\}} \mathord{\left/ {\vphantom {{\left\{ {{\upsigma }^{{2}}_{{{\text{Ap}}}} + {\upsigma }_{{{\text{ApAq}}}} } \right\}} {{\upsigma }^{{2}}_{{\text{A}}} }}} \right. \kern-\nulldelimiterspace} {{\upsigma }^{{2}}_{{\text{A}}} }},$$

2 $${\upgamma }_{{\text{q}}} = {{\left\{ {{\upsigma }^{{2}}_{{{\text{Aq}}}} + {\upsigma }_{{{\text{ApAq}}}} } \right\}} \mathord{\left/ {\vphantom {{\left\{ {{\upsigma }^{{2}}_{{{\text{Ap}}}} + {\upsigma }_{{{\text{ApAq}}}} } \right\}} {{\upsigma }^{{2}}_{{\text{A}}} }}} \right. \kern-\nulldelimiterspace} {{\upsigma }^{{2}}_{{\text{A}}} }}.$$

**Fig. 2:**
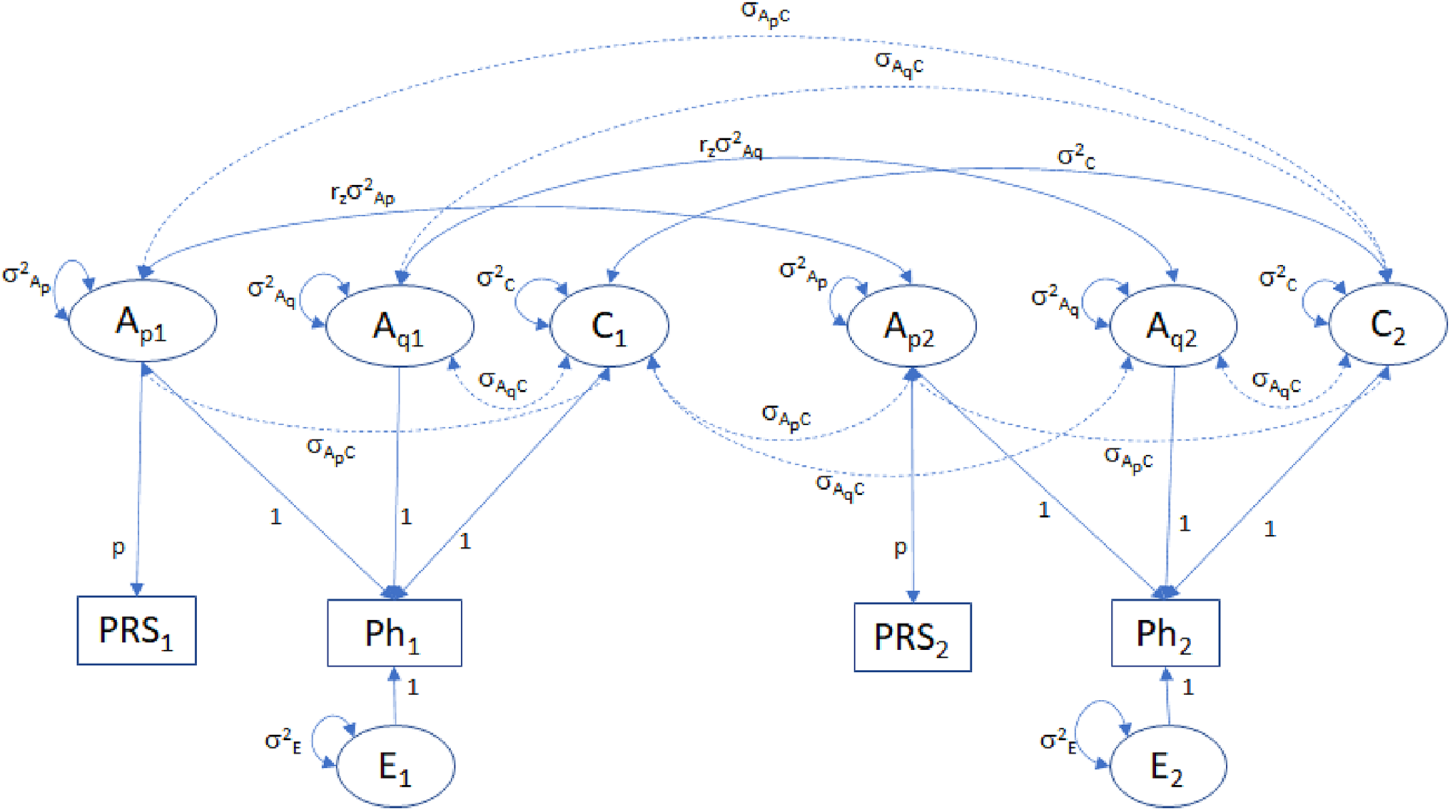
ACE twin model with PRSs, including A–C covariances σ_ApC_ and σ_AqC_ (dashed double headed arrows). This is the model in DZ twins (i.e., r_z_ = 0.5). The covariance between A_p_ and A_q_ is fixed to zero, but, as demonstrated in the text, this has no bearing on the derived estimate of the total A, C covarianc (σ_A,C_)

Note that σ_AC_ = σ_ApC_ + σ_AqC_, as γ_1_ + γ_2_ = 1. At this point two comments are in order. First, it is not possible to estimate both the scaling parameter p and the parameter σ_ApAq_. We therefore set σ_ApAq_ to equal zero. Given this identifying constraint, γ_p_ = σ^2^_Ap_/σ^2^_A_ and γ_q_ = σ^2^_Aq_/σ^2^_A_. We demonstrate below that the constraint σ_ApAq_ = 0 has no bearing on the likelihood ratio test of σ_AC_ = 0, or on the maximum likelihood estimates of σ_AC_, σ^2^_A_, and σ^2^_C_. Second, we recognize that if σ_AC_ ≠ 0, the PRS weights (b_l_) obtained in meta-analyses of the results of independent GWASs, which are used to calculate the PRS, will be upwardly (downwardly) biased given σ_AC_ > 0 (σ_AC_ < 0). This raises the question of whether this has any effect on the estimate of σ_AC_. We address this question below. The model is depicted in Fig. 2. We have parameterized this model in terms of variance components, i.e., we fixed the path coefficients terminating in the phenotype to one, and estimated the variance components (σ^2^_Ap_, σ^2^_Aq_, σ^2^_C_, σ^2^_E_, along with the parameter p). One may also consider fixing the variance components to one, and estimating the path coefficients. However, this parameterization may complicate statistical tests of the variance components (see Verhulst et al. 2019, for details).

## Simulation I: Power

In this model, the observed statistics are the 3 × 3 MZ (PRS, phenotype twin 1, phenotype twin 2) and the 4 × 4 DZ (PRS1, PRS2, phenotype twin 1, phenotype twin 2) covariance matrices (Σ_MZ_ and Σ_DZ_, respectively), and the 3- and 4-dimensional mean vectors. The MZ covariance matrix Σ_MZ_ is 3 × 3, as MZ twins, being genetically identical, have identical polygenic scores. We do not consider the mean structure of the phenotype data beyond noting that we adopt the standard (testable) assumptions that the means are equal over twins within a pair and over zygosity. Let the vector **θ** contain the six parameters of the covariance structure model:

$${\mathbf{\theta = }}\left[ {{\text{p}}\quad {\upsigma }^{2}_{{{\text{Ap}}}} \quad {\upsigma }^{2}_{{{\text{Aq}}}} \quad {\upsigma }^{2}_{{\text{C}}} \quad {\upsigma }^{2}_{{\text{E}}} \quad {\upsigma }^{2}_{{{\text{AC}}}} } \right].$$

The vector **θ** does not include σ_ApC_ and σ_AqC_ explicitly, because these parameters depend on the parameters σ^2^_Ap_, σ^2^_Aq_, and the total covariance σ_AC_, as shown above. We evaluated local identification numerically using the OpenMx function mxCheckIdentification (written by Michael Hunter). The model is locally identified if the Jacobian matrix, J(**θ**), is full column rank (Bekker et al. 1994). The Jacobian matrix contains the first-order derivatives of the non-redundant elements in the matrices Σ_MZ_(**θ**) and Σ_DZ_(**θ**) with respect to the parameters in **θ**. Given 6 + 10 elements in Σ_MZ_(**θ**) and Σ_DZ_(**θ**), and 6 parameters J(**θ**) is a 16 × 6 matrix. The mxCheckIdentification function is convenient as it does the necessary calculations automatically, and can be applied directly to the OpenMx script that one uses to fit the model.

Having established local identification, we proceeded to address the question of resolution by considering the statistical power to reject σ_AC_ = 0 given various parameter settings. We used exact data simulation (van der Sluis, et al. 2008), which is equivalent to analyzing the expected (true) covariance matrices. We used normal theory maximum likelihood estimation throughout, and based our power calculations on the non-centrality parameter (NCP) associated with the (non-central) chi-square distribution (Martin et al. 1978). Given exact data simulation, the NCP equals the loglikelihood ratio (LLR) test of σ_AC_ = 0. We used the OpenMx library (Boker et al. 2011; Neale et al. 2016) in the R program (R Core Team 2018), and we used R for data simulation, and power calculations. In the power analyses, we set the MZ and DZ sample sizes equal (Nmz = 1000, Ndz = 1000), and we report the power given Nmz = Ndz = 1000, and the required sample sizes to achieve a power of 0.80, given α = 0.05.

## Results I

The numerical check of model identification demonstrated that the model is locally identified, bearing in mind that we have set σ_ApAq_ = 0. That is, given the 3 × 3 MZ and the 4 × 4 DZ phenotypic covariance matrices, Σ_MZ_(**θ**) and Σ_DZ_(**θ**), we can obtain unique estimates of the six parameters p, σ^2^_Ap_, σ^2^_Aq_, σ^2^_C_, σ^2^_E_, and σ_AC_. From the perspective of the path model (Fig. 2), the key to the identification is the covariance between the phenotype and the PRS, which does not depend on zygosity. This covariance equals p*σ_Ap_^2^ + p*σ_ApC_ or p*γ_1_*(σ_A_^2^ + σ_AC_), where p, γ_1_ and σ_A_^2^ are identified based on the phenotypic and PRS MZ and DZ twin covariances. Table 1 contains the results of the power study. This table includes the 16 parameter settings and the power to reject σ_AC_ = 0.

**Table 1:** Statistical power to reject the null hypothesis that A–C covariance is zero (alpha = 0.05)

	*pr*PRS	*pr*Ph	σ^2^_A_	σ^2^_C_	σ^2^_E_	σ_AC_	r_AC_	σ^2^_Ph_	*pr*2σ_AC_	Power	N (0.80)
1	0.2	0.054	0.3 (0.27)	0.2 (0.18)	0.5 (0.45)	0.048	0.2	1.09	0.088	0.216	11,420
2	0.2	0.052	0.3 (0.26)	0.2 (0.17)	0.5 (0.43)	0.073	0.3	1.14	0.128	0.415	5158
3	0.2	0.053	0.3 (0.27)	0.3 (0.26)	0.4 (0.36)	0.060	0.2	1.12	0.107	0.323	6971
4	0.2	0.050	0.3 (0.25)	0.3 (0.25)	0.4 (0.34)	0.090	0.3	1.18	0.153	0.606	3161
5	0.4	0.109	0.3 (0.27)	0.2 (0.18)	0.5 (0.45)	0.048	0.2	1.09	0.088	0.402	5352
6	0.4	0.104	0.3 (0.26)	0.2 (0.17)	0.5 (0.43)	0.073	0.3	1.14	0.128	0.724	2408
7	0.4	0.107	0.3 (0.27)	0.3 (0.26)	0.4 (0.36)	0.060	0.2	1.12	0.107	0.595	3241
8	0.4	0.101	0.3 (0.25)	0.3 (0.25)	0.4 (0.34)	0.090	0.3	1.18	0.153	0.906	1462
9	0.2	0.088	0.5 (0.44)	0.2 (0.18)	0.3 (0.27)	0.063	0.2	1.12	0.112	0.238	10,107
10	0.2	0.084	0.5 (0.42)	0.2 (0.17)	0.3 (0.25)	0.094	0.3	1.18	0.159	0.455	4594
11	0.2	0.086	0.5 (0.43)	0.3 (0.26)	0.2 (0.17)	0.077	0.2	1.15	0.134	0.362	6084
12	0.2	0.081	0.5 (0.40)	0.3 (0.24)	0.2 (0.16)	0.116	0.3	1.23	0.189	0.661	2784
13	0.4	0.177	0.5 (0.44)	0.2 (0.18)	0.3 (0.26)	0.063	0.2	1.12	0.112	0.465	4481
14	0.4	0.168	0.5 (0.42)	0.2 (0.17)	0.3 (0.25)	0.094	0.3	1.18	0.158	0.794	2028
15	0.4	0.173	0.5 (0.43)	0.3 (0.26)	0.2 (0.17)	0.077	0.2	1.15	0.133	0.682	2650
16	0.4	0.162	0.5 (0.41)	0.3 (0.24)	0.2 (0.16)	0.116	0.3	1.23	0.188	0.950	1206

Given σ^2^_A_ = σ^2^_Ap_ + σ^2^_Aq_, *pr*PRS equals σ^2^_Ap_/σ^2^_A_, i.e., the proportion of additive genetic variance attributable to the PRS, and *pr*Ph is the proportion of phenotypic variance attributable to the PRS, σ^2^_Ap_/σ^2^_Ph_; r_AC_ and σ_AC_ are the correlation and covariance of A and C, σ^2^_Ph_ is the phenotypic variance; *pr*2*σ_AC_ is the proportion of phenotypic variance due to 2*σ_AC_

The standardized A, C, E variance components are given in parentheses. For instance, in setting 16, the raw variance is 0.5 + 0.3 + 0.2 + 0.116*2 = 1.23, and the standardized variance is 0.41 + 0.24 + 0.16 + 0.188 =  ~ 1

The power is given for Nmz = Ndz = 1000, given α = 0.05; N(0.80) is the sample size (N = Nmz + Ndz, where Nmz = Ndz) associated with a power of 0.80, given α = 0.05

Table 1 contains the proportions of genetic and phenotypic variance explained by the PRS (*pr*PRS and *pr*Ph in Table 1). These range from 0.2 to 0.4 (*pr*PRS) and 0.050 to 0.177 (*pr*Ph). The correlation between A and C (r_AC_) was chosen to equal 0.2 or 0.3. In addition to this correlation, we express the σ_AC_ effect size as the proportion (2σ_AC_)/σ^2^_Ph_ (i.e., *pr*2σ_AC_ in Table 1), where σ^2^_Ph_ = σ^2^_A_ + σ^2^_C_ + σ^2^_E_ + 2σ_AC_. This proportion ranges from 0.088 to 0.189. The proportion of A variance is ~ 0.26 or ~ 0.40; the proportion of C variance is ~ 0.18 or ~ 0.25, and the proportion of E variance varies between 0.09 and 0.44). The settings, which are limited, were chosen merely to identify some circumstance in which the power to reject σ_AC_ = 0 is acceptable, given the present sample sizes.

**Fig. 3:**
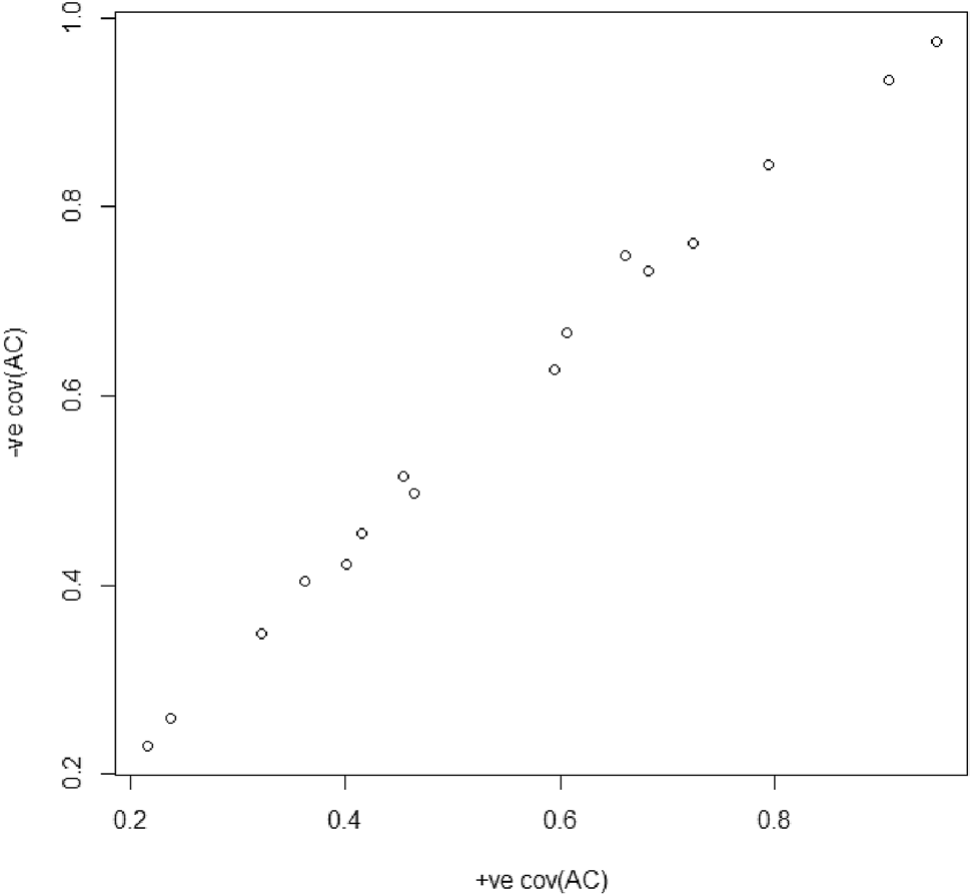
Power of the LLR test to reject σ_AC_ = 0 given positive and negative σ_AC_. The parameter settings are given in Table 1. The only difference is the sign of σ_AC_. The power to reject σ_AC_ = 0 given positive σ_AC_ is given in Table 1

The greatest power is obtained in settings 8, 14, and 16: 0.906 (8), 0.793 (14), and 0.950 (16). Here, the PRS accounts for 10.1% (8), 16.8% (14), and 16.2% (16) of the phenotypic variance, and 2σ_AC_ accounts for 15.3% (8), 15.9% (14), and 18.9% (16) of the phenotypic variance. We see the lowest power given settings 1 (0.216) and 9 (0.238). Unsurprisingly, these are associated with relative low values of *pr*2σ_AC_ (8.8% and 11.2%) and *pr*Ph (5.4% and 8.8%). The relative contributions of *pr*2σ_AC_ and *pr*Ph to the power are apparent in the correlations of these with the power: 0.71 and 0.53, respectively. Regressing the power on these parameters, we found that they explain 65% of the variance in power (β *pr*2σ_AC_: 0.625 and β *pr*Ph 0.400). Both are important, but the contribution of *pr*2σ_Ac_ to the power is greater. The comparison of the σ^2^_A_ = 0.3 and the σ^2^_A_ = 0.5 conditions show that the magnitude of σ^2^_A_ does not greatly influence the power. In terms the ratio of the N (power = 0.80) are about 1.1–1.2 (favoring the σ^2^_A_ = 0.5 conditions). Finally, to determine the effect of the sign of σ_AC_, we repeated the power analysis with r_AC_ set to equal − 0.2 or − 0.3, and all other parameters unchanged. Figure 3 displays the plot of the power given positive and negative r_AC_. We note that the difference in power is small suggesting that the sign of rAC is unimportant in calculating power.

## Simulation II: Bias and Type I Error Rate

As noted above, the weights (b_l_) used to calculate the PRS are expected be biased upwards if σ_AC_ > 0. We investigated the effects of this in a simulation study. Specifically, we simulated data according to the nuclear twin family (NTF) design (Fulker 1988; Keller et al. 2009), which comprises MZ and DZ twins and their parents. This model includes cultural transmission, i.e., the direct contribution of the parental phenotype to the twin's environment (parameter m in the notation of Keller et al. 2009 notation). This contribution gives rise to a shared environmental variable (F in the notation of Keller et al. 2009), and to covariance between this variable (F) and the additive genetic factor (A), σ_AF_. In addition to the shared variance due to F, the model includes a shared environmental variance term, due to shared influences other than cultural transmission. We denote this C* to distinguish it from the C in the standard ACE model. The original NTF model accommodates phenotypic assortative mating. However, here we assume that mating is random. The MZ and DZ expected phenotypic covariance matrices are shown in Table 2. It is not possible to resolve F and C* in the absence of parental phenotypes. Thus, in the standard ACE model and in the model with PRSs, we estimate a single shared environmental variance, σ^2^_C_ which actually equals σ^2^_C*_ + σ^2^_F_.

**Table 2:** The expected covariance matrices in simulations 1–3

	MZ 1	MZ 2
MZ 1	σ^2^_A_ + σ^2^_C*_ + σ^2^_F_ + 2σ_AF_ + σ^2^_E_	σ^2^_A_ + σ^2^_C*_ + σ^2^_F_ + 2σ_AF_
MZ 2	σ^2^_A_ + σ^2^_C*_ + σ^2^_F_ + 2σ_AF_	σ^2^_A_ + σ^2^_C*_ + σ^2^_F_ + 2σ_AF_ + σ^2^_E_
	DZ 1	DZ 2
DZ 1	σ^2^_A_ + σ^2^_C*_ + σ^2^_F_ + 2σ_AF_ + σ^2^_E_	½σ^2^_A_ + σ^2^_C*_ + σ^2^_F_ + 2σ_AF_
DZ 2	½σ^2^_A_ + σ^2^_C*_ + σ^2^_F_ + 2σ_AF_	σ^2^_A_ + σ^2^_C*_ + σ^2^_F_ + 2σ_AF_ + σ^2^_E_
	σ_F_^2^ = 2m^2^(σ^2^_A_ + σ^2^_C*_ + σ^2^_F_ + 2σ_AF_+ σ^2^_E_) = 2m^2^σ^2^_Ph_	
	σ_AF_ = (mσ_A_)/(1−σ_F_ m)	

Parameter m is the regression coefficient in regression of parental phenotype on F in twins (shared environmental factor attributable to cultural transmission)

σ^2^_F_ is shared environmental variance due to cultural transmission (see Keller et al. 2009, for the derivation)

σ^2^_C*_ is shared environmental variance, not due to cultural transmission

σ^2^_E_ is unshared environmental variance

σ^2^_A_ additive genetic variance

σ_AF_ covariance of A and F (see Keller et al. 2009, for the derivation)

Note in fitting the model we estimate σ^2^_C_ (i.e., σ^2^_F_ + σ^2^_C*_)

First, we considered the model without cultural transmission, i.e., cultural transmission parameter (m) was zero. This implies that σ^2^_F_ and σ_AF_ are zero, as the parameter m is the source of the A–C covariance. However, we included σ^2^_C*_ > 0. Thus, there is shared (by the twins) environmental variance, but it is not due to cultural transmission. This model allows us to determine the Type I error rate associated with the test of σ_AC_ = 0. As an aside, in this simulation, the inclusion of σ^2^_C*_ > 0 also allows us to establish the power to detect C variance in a twin model with PRS, in addition to checking the Type I error rate in test of σ_AC_ = 0. Second, we considered a model with cultural transmission, with m > 0, so that σ_AF_ > 0 and σ^2^_F_ > 0. In this model we set σ^2^_C*_ to zero, so that there are no shared environmental influences other than those stemming from the cultural transmission. Third, we considered a model with cultural transmission (m > 0), so that σ_AF_ > 0 and σ^2^_F_ > 0, and σ^2^_C*_ > 0. As mentioned, we cannot resolve σ^2^_F_ and σ^2^_C*_, so we fitted a single shared environmental factor, representing C and F. In summary, given σ^2^_C_ = σ^2^_F_ + σ^2^_C*_, we have the following settings. Simulation 1: m = 0, σ^2^_F_ = 0, σ^2^_C*_ > 0, σ^2^_C_ = σ^2^_C*_, σ_AC_ = σ_AF_ = 0. Simulation 2: m > 0, σ^2^_F_ > 0, σ^2^_C*_ = 0, σ^2^_C_ = σ^2^_F_, σ_AC_ = σ_AF_ and σ_AF_ > 0. Simulation 3: m > 0, σ^2^_F_ > 0, σ^2^_C*_ > 0, σ^2^_C_ = σ^2^_F_ + σ^2^_C*_, σ_AC_ = σ_AF_ and σ_AF_ > 0.

In each simulation study based on these three models, we carried out 500 replications. Each data set comprised genotypic and phenotypic data in parents and twins. The parental data were discarded, and the twin data were used to fit the model. The additive genetic variable comprised 100 uncorrelated diallelic genetic variants, of which 40 were used to calculate the PRS. We carried out the simulation twice: once with exact, unbiased PRS weights (i.e., the parameters b in the expression PRS_i_ = ${\sum }_{\mathrm{l}=1}^{\mathrm{L}}{\mathrm{b}}_{\mathrm{l}}$ GV_li_), and once with estimated weights b. We estimated the weights in independent data (not the data used to fit the actual model) by regressing the phenotype on the genetic variants. Given the absence of A–C covariance, the estimated weights are unbiased. In each replication the sample sizes were N = 2000 to estimate the parameter weights b, and Nmz = 1000 and Ndz = 1000 (in total 2000 pairs) to fit the actual model. Parameter values and effect sizes are given in Table 3. In simulations 1–3, the true values of *pr*Ph are 0.2, 0.141, and 0.147, and the true values of *pr*2σ_A,C*+F_ are 0.0, 0.353, and 0.368, respectively.

**Table 3:** Means and standard deviation of parameter estimates in simulation 1–3 based on 500 replications (Nmz = 1000; Ndz = 1000)

	b est	σ^2^_Ap_	σ^2^_Aq_	σ^2^_C*_	σ^2^_F_	σ^2^_C_** = ** σ^2^_C*_ + σ^2^_F_	σ^2^_E_	σ_A,C_
Simulation 1								
True		**0.20**	**0.30**	**0.20**	**0**	**0.20**	**0.30**	**0.00**
Mean	No	0.199	0.298	0.197	–	–	0.301	0.003
s.d.		0.026	0.045	0.058	–	–	0.013	0.033
s.e.(mean)		0.0012	0.0020	0.0026			0.0006	0.0015
Mean	Yes	0.184*	0.316*	0.199	–	–	0.300	0.001
s.d.		0.026	0.047	0.063	–	–	0.013	0.036
s.e.(mean)		0.0012	0.0021	0.0028			0.0006	0.0016
Simulation 2								
True		**0.20**	**0.30**	**0**	**0.091**	**0.091**	**0.30**	**0.125**
Mean	No	0.200	0.300	–	0.087	–	0.301	0.126
s.d.		0.026	0.047	–	0.078	–	0.013	0.037
s.e.(mean)		0.0012	0.0021		0.0035		0.0006	0.0017
Mean 2	Yes	0.189*	0.315*	–	0.090	–	0.300	0.124
s.d.		0.025	0.046	–	0.079	–	0.013	0.038
s.e.(mean)		0.0011	0.0021		0.0035		0.0006	0.0017
Simulation 3								
True		**0.20**	**0.30**	**0.20**	**0.108**	**0.308**	**0.30**	**0.125**
Mean	No	0.200	0.302	–	–	0.302	0.300	0.126
s.d.		0.026	0.045	–	–	0.077	0.013	0.039
s.e.(mean)		0.0012	0.0020			0.0034	0.0006	0.0017
Mean	Yes	0.185*	0.320*	–	–	0.304	0.299	0.125
s.d.		0.026	0.050	–	–	0.087	0.013	0.041
s.e.(mean)		0.0012	0.0022			0.0039	0.0006	0.0018
Simulation 2^a^								
True		**0.20**	**0.30**	**0**	**0.091**	**0.091**	**0.30**	**0.125**
Mean	Yes	0.189*	0.314*	–	0.095	–	0.299	0.122
s.d.		0.025	0.045	–	0.061	–	0.013	0.032
s.e.(mean)		0.0011	0.0020		0.0027		0.0006	0.0014

Values shown in bold are the true parameter values

Simulation 2^a^: subject to constraints of positive definiteness of the A_p_–C and A_q_–C covariance matrices

b est: weights for PRS estimated (yes), or fixed to true values (no)

Simulation 1: r(A,F + C) = 0; σ^2^_Ph_ = 0.20 + 0.30 + 0.20 + 0.30 = 1; r(MZ) = 0.70 & r(DZ) = 0.45; *pr*PH = 0.2; *pr*2σ_AC_ = 0.0

Simulation 2: r(A,F + C) = 0.125/sqrt(0.5*0.091) = 0.586; σ^2^_Ph_ = 1.141; r(MZ) = 0.74 & r(DZ) = 0.52; *pr*PH = 0.141; *pr*2σ_AC_ = 0.353

Simulation 3: r(A,F + C) = 0.125/sqrt(0.5*0.308) = 0.318; σ^2^_Ph_ = 1.358; r(MZ) = 0.78 & r(DZ) = 0.59; *pr*PH = 0.147; *pr*2σ_AC_ = 0.368

*Deviation from true value is significant given α = 0.01

Note in fitting the model we estimated the single variance term σ^2^_C_, which equals σ^2^_F_ + σ^2^_C*_. In simulations 1, σ^2^_F_ is zero and σ_AC_ = 0; in simulation 2 σ^2^_C*_ is zero, σ_AC_ > 0; in simulation 3, σ^2^_F_ > 0, σ^2^_C*_ > 0, and σ_AC_ > 0

In summary, the aims were (1) to establish that the Type 1 error rate was correct and (2) to investigate the effects, if any, of biased weights on the Type I error rate and the parameter estimates (bias), (3) to determine whether the presence of PRS, given zero A–C covariance, increases the power to detect C variance.

## Results II

We first discuss the parameter estimates and then the loglikelihood ratio (LLR) test statistics. Table 3 contains the true parameter values and the mean and standard deviation of the parameter estimates based on the 500 replications. In simulation 1, given exact PRS weights, the parameter estimates are unbiased, as expected. Given estimated PRS weights, the estimates of σ^2^_Ap_ and σ^2^_Aq_ are biased: the mean values are 0.184 (underestimated; true 0.2) and 0.316 (overestimated; true: 0.3), respectively. The estimate of the covariance term σ_AC_ is unbiased: the mean value is 0.001 (true: 0.0). In simulation two, given exact weights, the parameter estimates are again unbiased, and given estimated PRS weights, the estimates of σ^2^_Ap_ and σ^2^_Aq_ are again biased: the mean values are 0.189 (underestimated; true 0.2) and 0.315 (overestimated; true: 0.3), respectively. The estimate of σ_AC_ is unbiased: mean value 0.124 (true 0.125). Simulation three produced the same results as simulation two, in terms of parameter bias stemming from using estimated PRS weight. The main finding is that using estimated PRS weights results in biased estimates of the variance components σ^2^_Ap_ and σ^2^_Aq_, but has little effect on the estimate of the covariance term σ_AC_.

Table 4 contains the results of the LLR tests. We tested the hypotheses σ_A,C_ = 0, and σ^2^_C_ = 0 given σ_A,F+C_ = 0 in the twin model with the PRSs. In addition, we tested the hypothesis σ_C_^2^ = 0 in the standard univariate ACE model. The test of σ^2^_C_ = 0 is of interest in simulation 1, as the comparison of σ^2^_C_ = 0 given σ_A,F+C_ = 0 (in the full model) and hypothesis σ^2^_C_ = 0 in the standard univariate ACE model tell us whether the presence of PRSs helps to resolve C. The LLR statistic associated with the test of σ_A,F+C_ = 0 in simulation 1, where in truth σ_A,F+C_ = 0, should follow central chi2(1) distribution, which is characterized by a mean of 1 and a standard deviation of √(2) = 1.414. Given exact PRS weights, the mean and standard deviation of the LLR statistic are 0.970 and 1.423 (see Table 4). These do not differ from the expected values of 1 and √2 (LLR test: 0.27, df = 2, p = 0.87). The Type I error rate equaled 0.049 (CI95: 0.032–0.072). Given estimated weights, the values are 1.041 and 1.515. While these do not appear to deviate from the expected values (LLR test: 5.18, df = 2, p = 0.075), the variance is larger (1.515 vs. 1.414), as is the Type I error rate: 0.063 (CI95: 0.043–0.088). In terms of the mean LLR test, we note that the test of σ_C_^2^ = 0 is more powerful in the full model (given σ_A,C_ = 0) than in the standard univariate ACE twin model. With exact PRS weights, the mean LLR test statistics equal 22.2 (with PRSs) and 16.06 (standard ACE model), and with estimated PRS weight, 21.5 and 15.6.

**Table 4:** Means and standard deviation of loglikelihood ratio tests simulation 1–3 based on 500 replications (Nmz = 1000; Ndz = 1000)

	b est	σ_A,C_ = 0	σ^2^_C_ = 0 given σ_A,C_ = 0	σ^2^_C_ = 0 in ACE model
Simulation 1				
Mean	No	0.970^a^	22.2	16.06
sd		1.423	8.88	7.344
Mean	Yes	1.041^a^	21.52	15.62
sd		1.515	8.94	7.49
Simulation 2				
Mean	No	15.11	25.12	37.16
sd		7.47	10.13	11.23
Mean	Yes	13.51	25.99	36.63
sd		7.38	9.66	11.25
Simulation 3				
Mean	No	13.48	78.87	78.98
sd		7.16	15.40	15.96
Mean	Yes	11.96	74.59	78.43
sd		6.65	16.93	17.05
Simulation 2^b^				
Mean	Yes	13.92	25.54	36.66
sd		7.49	9.92	12.11

Means are the mean of the 1-df likelihood ratio test

b est: weights for PRS estimated (yes), or fixed to true values (no)

Simulation 1: r(A,C) = 0; σ^2^_Ph_ = 0.20 + 0.30 + 0.20 + 0.30 = 1; r(MZ) = 0.70 & r(DZ) = 0.45; *pr*PH = 0.2; *pr*2σ_AC_ = 0.0

Simulation 2: r(A, C) = 0.125/sqrt(0.5*0.091) = 0.586; σ^2^_Ph_ = 1.141; r(MZ) = 0.74 & r(DZ) = 0.52; *pr*PH = 0.141; *pr*2σ_AC_ = 0.353

Simulation 3: r(A,C) = 0.125/sqrt(0.5*0.308) = 0.318; σ^2^_Ph_ = 1.358; r(MZ) = 0.78 & r(DZ) = 0.59; *pr*PH = 0.147; *pr*2σ_AC_ = 0.368

^a^Expected mean value = 1, expected stdev = √2 = 1.414

^b^Subject to constraints of positive definiteness of the A_p_–C and A_q_–C covariance matrices

The results of simulation 2 and 3 are comparable. The test of σ_AC_ = 0 suffers slightly given estimated PRS weights: the mean LLR test statistics equal 15.10 and 13.5 (simulation 2) and 13.48 and 11.96 (simulation 3). The effect of the test of σ^2^_C_ = 0 given σ_AC_ = 0 is slight (in simulation 3: 78.8 vs 74.6). We note that the test of C in the standard twin model appears to be more powerful. However, given σ_AC_ > 0, this is a combined test of σ_C_^2^ = 0 and σ_AC_ = 0.

**Fig. 4:**
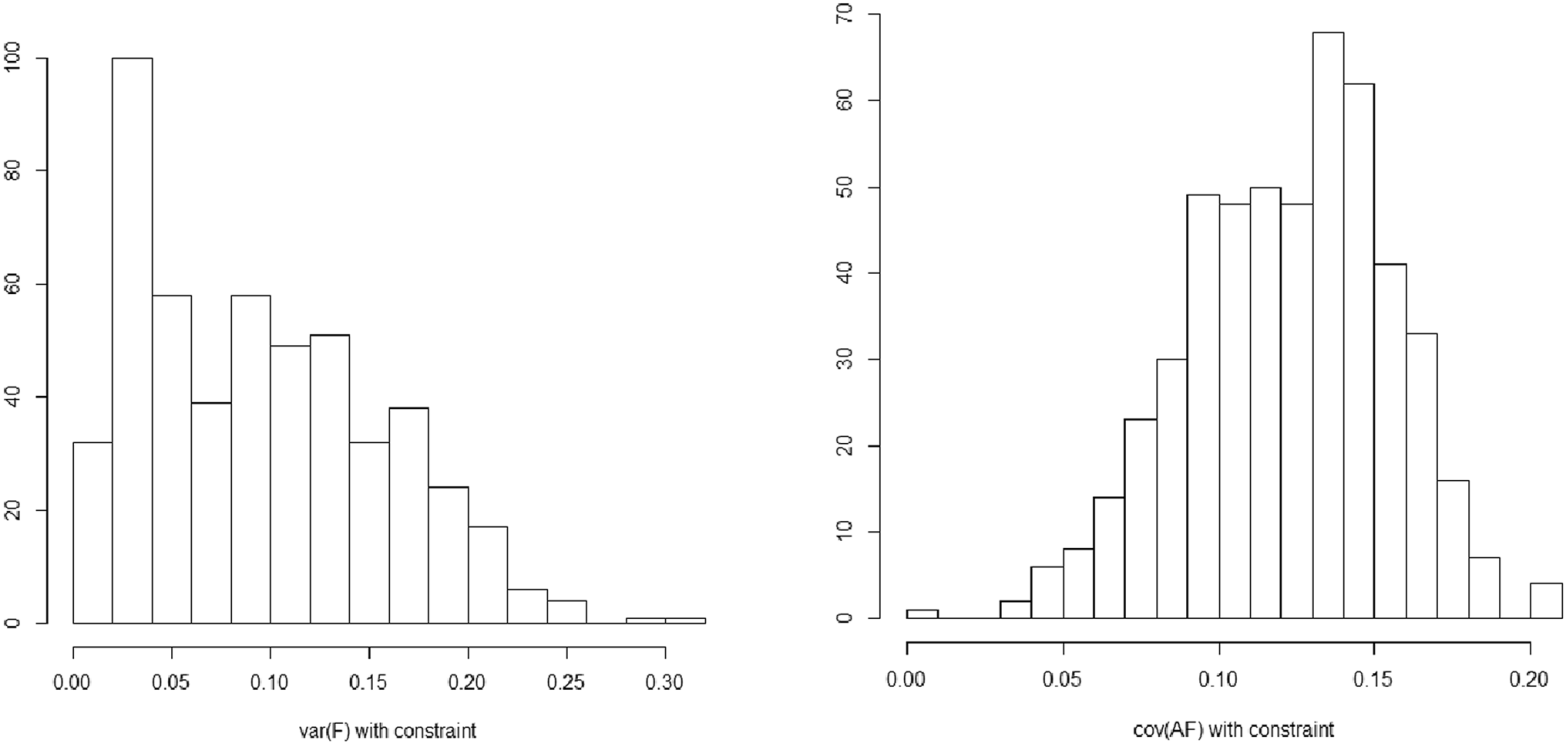
Distribution of estimates of σ^2^_F_ (left) and σ_AF_ (right) given positive definiteness constraints (simulation 2). The true values are σ^2^_F_ = 0.091 and σ_AF_ = 0.125

In simulation 2, we set σ^2^_C*_ = 0 and σ^2^_F_ = 0.091 (6.4% of the phenotypic variance). This relatively low value does not rule out a considerable contribution of σ_AC_ to the phenotypic variance (35%). The true twin correlations in the simulation 2 (r_MZ_ = 0.74, r_DZ_ = 0.52) suggest a considerable contribution of C (~ 30%). This implies that (1) substantial C in the classical twin model may be mainly due to A–C covariance, while (2) the C variance, which in simulation 2 comprised only σ^2^_F_, may be small. Given that this variance may be small, its estimate may, given the present variance component parameterization, assume negative values. Indeed, in simulation 2, we encountered a negative variance component estimate in about 11% (exact PRS weights) and 13% (estimated weights) of the replications (we did not remove these cases in calculating the results in Tables 3 and 4 to avoid the bias caused by truncating the distribution of the parameters to the admissible solutions). The problem of negative variance can be solved by imposing the constraint that the 2 × 2 covariance matrix of A_p_ and C and the 2 × 2 covariance matrix of A_q_ and C be positive definite. This means that the eigenvalues of the covariance matrices are constrained to be larger than zero. This PD constraint is simple to implement in OpenMx using an mxAlgebra statement, as OpenMx includes a function to calculate eigenvalues (see the OpenMx script). Only constraining σ^2^_C_ to be greater than zero is insufficient, as this by itself does not ensure that the covariance matrix of A_p_ (or A_q_) and C is positive definite. In addition, if σ^2^_C_ were to hit the lower bound, the parameter σ_AC_ would no longer be defined. We repeated simulation 2 with these PD constraints to gauge the effects on the parameters. The results, as obtained with estimated PRS weights, are included in Tables 3 and 4. These results are largely consistent with those obtained without the PD constraints. We do note that the PD constraints affect the distribution of the estimates of σ^2^_C_ positively skewed, as shown in Fig. 4. Without the positive definiteness constraints, this distribution is normal. In contrast, the distribution of the estimates of σ_AC_ appear to be quite normal, which suggests that, at least in the present scenario, the LLR test of σ_AC_ = 0 will not be affected by the PD constraints.

## The Identifying Constraint σ_ApAq_ = 0

As mentioned above, we have constrained σ_ApAq_ to equal 0, because it is not possible to estimate the scaling parameter p (in σ^2^_PRS_ = p^2^σ^2^_Ap_) and the covariance simultaneously. This raises the question how the results are affected if in fact σ_ApAq_ > 0, as σ_ApAq_ = 0 is implausible given linkage disequilibrium. In fact, the constraint σ_ApAq_ = 0 has no effect of the estimates of σ_AC_, σ^2^_A_, and σ^2^_C_. To demonstrate this, we used simulation exact data with σ_ApAq_ > 0, and fitted the model twice. Once with σ_ApAq_ fixed to its true value, and once σ_ApAq_ fixed to zero. Specifically, we chose the parameter values shown in Table 5. Fitting the model with σ_ApAq_ fixed to equal its true value (σ_ApAq_ = 0.1224; correlation: ρ_ApAq_ = 0.5), we recovered the parameter values, including σ_AC_ = 0.077, and the total additive genetic variance 0.2 + 0.3 + 2*0.1224 =  ~ 0.745 (i.e., σ^2^_Ap_ + σ^2^_Aq_ + 2σ_ApAq_). The power to reject σ_AC_ = 0 equals 0.742 (α = 0.05). Fixing σ_ApAq_ = 0, we obtain identical results, except for the values of σ^2^_Ap_ and σ^2^_Aq_. The total genetic variance is now composed as follows 0.5199 + 0.2250 = 0.745 (i.e., σ^2^_Ap_ + σ^2^_Aq_). We checked this result with a wide variety of parameter values. So in principle, one can constrain σ_ApAq_ to equal any sensible value. However, while the estimates and tests of σ_AC_, σ^2^_A_, and σ^2^_C_ are unaffected, we note that the values of σ^2^_Ap_ and σ^2^_Aq_ do depend on this sensible value.

**Table 5:** Results with σ_ApAq_ fixed to its true value (row A), and σ_ApAq_ fixed to equal zero (row B)

	σ^2^_Ap_	σ^2^_Aq_	σ_ApAq_	σ^2^_A_	σ^2^_C_	σ^2^_E_	σ_AC_	LLR	Power
A	0.20	0.30	0.1224*	0.745^a^	0.2	0.3	0.077	6.81	0.742
B	0.5199	0.2250	0*	0.745^b^	0.2	0.3	0.077	6.81	0.742

σ_ApAq_ = 0.122 corresponds to a correlation of 0.1224/sqrt(0.20*0.30) = 0.5

σ_AC_ = 0.077 corresponds to a correlation of 0.077/sqrt(0.745*0.2) = 0.2

Power: to reject σ_AC_ = 0 is given α = 0.05, and Nmz = 1000, Ndz = 1000, based on the LLR statistic

*Fixed parameters

^a^σ^2^_A_ = 0.745 = 0.30 + 0.30 + 2*0.1224

^b^σ^2^_A_ = 0.745 = 0.5199 + 0.2250

## Discussion

The present aim was to estimate A–C covariance in the classical twin model with PRS. To this end, we proposed the model depicted in Fig. 1, in which the covariance between A_p_ (PRS) and A_q_ and C are modeled as a function of the single covariance of A (A_p_ + A_q_) and C. We found that the power to reject σ_AC_ = 0 depends mainly on the proportion of phenotypic variance due to the covariance term (σ_AC_) and the PRS, where the former is more important than the latter. We investigated the influence of using estimated PRS weights. The use of estimated weights resulted in downwards bias of σ_Ap_ and an upwards bias of σ_Aq_. However, the estimate of σ_AC_ was not affected. The use of estimated weights had a small effect of the Type I error rate in the test of σ_AC_ = 0.

In the most favorable settings qua power (8, 14, and 16 in Table 1), the proportions *pr*2σ_AC_ (phenotypic variance due to σ_AC_) equaled 0.153, 0.159, and 0.189, and the proportions *pr*Ph (phenotype variance due to the PRS) equaled 0.101, 0.168 and 0.162. We consider these values (*pr*Ph) to be generally large by today's standards, but note that, while *pr*2σ_AC_ is given, the proportion *pr*Ph is likely to increase with the ongoing progress of GWAS meta analyses of many phenotypes. For instance, at present PRSs explain ~ 15% of the variance of educational attainment and ~ 11% of the variance of IQ (Allegrini et al. 2019).

The results of the power study and simulations shed some light on the viability of the model. But the results of simulation 2 also demonstrated that positive cultural transmission can result in large C in the standard ACE model, while most of this C variance is due to σ_AC_. The actual C variance (without σ_AC_) can be quite small. It is therefore advisable to fit the model with the positive definiteness constraint, outlined above. In this connection, we note that the finding that C variance in cognitive abilities is large in young children, but declines quickly in magnitude as children grow older (Haworth et al. 2010; Tucker-Drob and Bates 2016) may well be due to a decline in the magnitude of cultural transmission, in combination with an increase in genetic variance. This may be testing by extending the present model to include age as a moderator in the manner of Purcell (2002). This is relatively simple to do in OpenMx. In this connection, we also note that the estimate of σ_AC_ obtained using the present model may tell us that σ_AC_ is present. It does not, unlike other models, reveal the source of the σ_AC_. For instance, in the NTF design, cultural transmission is the source the covariance between A and F, where the distinction is made between F (shared environmental effects due to cultural transmission) and residual C, which we denoted C^*^ above.

We considered negative σ_AC_ in the power study, and found that the power to reject σ_AC_ = 0 was about the same regardless of the sign of σ_AC_. We note that negative σ_AC_ (e.g., originating in negative cultural transmission) tends to produce twin correlations, which are suggestive of an ADE model (2*r_DZ_ < r_MZ_). This is to be expected as − σ_AC_ lowers the MZ and DZ correlations to the same extent. Finally, the present results demonstrated that the addition of PRSs to the ACE model increases the power to detect C variance, assuming σ_AC_ = 0. This may be of interest, as in the classical twin model, the power to detect C variance is known to be generally poor (Visscher et al. 2008; Martin et al. 1978).

In closing, we note the following limitations. We have assumed that dominance variance (D) is absent, and acknowledge that the twin univariate design is limited to ACE or ADE. As demonstrated by Keller et al. (2010), a well fitting ACE model does not rule out the represent of D. It is possible that the addition of PRSs may aid in resolving D (in an ACDE) model, but we consider this beyond the present scope. Boomsma et al (2020; see this issue) showed that it is possible to estimate all four variance components (A, C, D, and E) in special cases of the multivariate twin model. Second as mentioned above, the settings of the power study and the simulations are limited in scope. In addition, we considered only equal MZ and DZ sample sizes (Nmz = Ndz = 1000). The ratio Nmz/Ndz has a general bearing on the power in the twin design (Visscher 2004). However, power calculations with unequal MZ and DZ sample sizes are simple to carry out. Third, the simulations that we carried out to gauge the effect of using estimated PRS weights, involved only a small number of associated GVs with relatively large effects. Simulation studies with more realistic designs will provide additional information concerning the effects of using estimated PRS weights. Fourth, we assumed that phenotypic mating is random. However, we note that the PRSs in the DZ twins offer the means to tests this, as the correlation between the additive genetic PRSs will equal 0.5 given phenotypic random mating. In addition, the present model may be extended to include parental data to accommodate phenotypic assortative mating as outlined in Keller et al. (2009). Fifth, we have not considered the effect of violations of other standard twin design assumptions on the estimate of σ_AC_ (Eaves et al. 1977; Purcell 2002; Keller et al 2010). Genotype—unshared environment covariance (σ_AE_) is not identified in the present model. Unmodeled (positive) σ_AE_ and A × C interaction contribute to A. We do not see how either could result in spurious σ_AC_. A × E and C × E interaction contribute to E, which has no bearing on A, C, or σ_AC_. Sixth, we have not considered the possibility that the σ_AC_ is due to stratification. We know that spatial (geographical) allele frequency gradients may given rise to spurious C variance in the classical twin design (Tamimy et al. 2020; see this issue). A positive spatial correlation between C effects and allele frequencies, may given rise to A–C covariance. One way to detect this by including as fixed covariates principal components that reflect the allele frequency gradient (Price et al. 2006). If this kind of stratification is an issue, then the size of the C variance (see Tamimy et al. 2020) and the size of the A–C covariance should decline following the introduction of these covariates. Finally, we have assumed that the PRSs weights were obtained from GWASs of the phenotype of interest. Whether the present approach can be adapted to handle PRSs weights based on a genetically correlated phenotype (i.e., correlated with the phenotype of interest) remains to be seen.

## Supplementary information

Below is the link to the electronic supplementary material.

Supplementary material 1 (DOCX 22 kb)
