# Supplementary material for: Incorporating Polygenic Risk Scores in the ACE Twin Model to Estimate A–C Covariance
Source: Behav Genet. 2021 Feb 1;51(3):237–49. doi: 10.1007/s10519-020-10035-7 (PMC8093156; doi:10.1007/s10519-020-10035-7)
Supplement: Supplementary file 1 — Supplementary material 1 (DOCX 22 kb) [file 10519_2020_10035_MOESM1_ESM.docx]

**OpenMx R code.**

The covariance matrices are calculated as follows (in the notation of the OpenMx code):

Smz = L latSmz L^t^

Sdz = L latSdz L^t^

where the matrix (4x8) matrix L (named "LY" in the OpenMx code) and the (8x8) matrices latSmz and latSdz are defined in the OpenMx code. The starting values provided in the OpenMx code below will require careful consideration in any application.

# --------------------------------------------------- start

rm(list=ls(all=TRUE)) # clear working memory

#

library(OpenMx) # OpenMx

library(MASS) # needed to simulate data

# We use the NPSOL optimizer. The default optimizer can also be used

mxOption(NULL, "Default optimizer","NPSOL")

#

# the following artificial data are included

# merely to run the model

#

# MZ Means

mmz=c(8,0,8,0)

names(mmz)=c('T1','p1','T2','p2')

# MZ covariance matrix

Smz=matrix(c(

1.2323790, 0.2464758, 1.0323790, 0.2464758,

0.2464758, 0.2000000, 0.2464758, 0.2000000,

1.0323790, 0.2464758, 1.2323790, 0.2464758,

0.2464758, 0.2000000, 0.2464758, 0.2000000),4,4,byrow=T)

rownames(Smz)=colnames(Smz)=c('T1','p1','T2','p2')

#

# DZ Means

mdz=c(8, 0, 8, 0)

names(mdz)=c('T1','p1','T2','p2')

#DZ Covariance matrix

Sdz=matrix(c(

1.2323790, 0.2464758, 0.7823790, 0.1464758,

0.2464758, 0.2000000, 0.1464758, 0.1000000,

0.7823790, 0.1464758, 1.2323790, 0.2464758,

0.1464758, 0.1000000, 0.2464758, 0.2000000),4,4,byrow=T)

rownames(Sdz)=colnames(Sdz)=c('T1','p1','T2','p2')

vnames=c('T1','p1','T2','p2')

#

# sample size in pairs

NMZ=nmz=1000

NDZ=ndz=1000

#

S2mz= (Smz[1:3,1:3]) # discard PRS of second MZ twin

mmz=mmz[1:3]

S2dz= (Sdz[1:4,1:4])

#

# simlulate data using mvrnorm()

dmz=matrix(0,NMZ,3)

dmz[,1:3]=mvrnorm(NMZ,mmz,Sigma=S2mz,emp=T)

ddz=mvrnorm(NDZ,mdz,Sigma=S2dz,emp=T)

colnames(ddz)=vnames

colnames(dmz)=vnames[1:3]

ddz=as.data.frame(ddz)

dmz=as.data.frame(dmz)

#

# we now have data to fit the model

# OpenMx code ........................

# variable names ..

vnames=c('T1','p1','T2','p2')

#

part1 <- mxModel(model="PARS",

#

mxMatrix(type="Full", nrow=4, ncol=8,

free=matrix(c(

F,F,F,F,F,F,F,F,

F,T,F,F,F,F,F,F,

F,F,F,F,F,F,F,F,

F,F,F,F,F,T,F,F),4,8,byrow=T),

values=matrix(c(

1,1,1,1,0,0,0,0,

0,.9,0,0,0,0,0,0, # st values .9

0,0,0,0,1,1,1,1,

0,0,0,0,0,.9,0,0),4,8,byrow=T),

label=matrix(c(

NA,NA,NA,NA,NA,NA,NA,NA,

NA,"p",NA,NA,NA,NA,NA,NA, # p is the scaling parameter

NA,NA,NA,NA,NA,NA,NA,NA,

NA,NA,NA,NA,NA,"p",NA,NA),4,8,byrow=T), name="LY"),

#

mxMatrix(type="Full", nrow=1, ncol=1, free=TRUE, values=.2, label="sA1", name="SA1"), # variance of PRS (Ap)

mxMatrix(type="Full", nrow=1, ncol=1, free=TRUE, values=.4, label="sA2", name="SA2"), # residual A variance (Aq)

mxMatrix(type="Full", nrow=1, ncol=1, free=TRUE, values=.3, label="sC", name="SC"), # variance of C

mxMatrix(type="Full", nrow=1, ncol=1, free=TRUE, values=.8, label="sE", name="SE"), # variance of E

mxMatrix(type="Full", nrow=1, ncol=1, free=FALSE, values=.0, label="rAP", name="r12"), # assumption.....

mxMatrix(type="Full", nrow=1, ncol=1, free=TRUE, values=.0, label="cAC", name="CAC"), # cov(AC)

mxMatrix(type="Full", nrow=2, ncol=1, free=FALSE, values=0, name="z21"), # zero matrix for constraints

#

# get weights for Cov(A1,C) Cov(A2,C)

# r12 = 0

mxAlgebra(SA1 + SA2 + 2*r12*sqrt(SA1*SA2), name="SA"),

#

mxAlgebra(r12*sqrt(SA1)*sqrt(SA2), name="C12"),

mxAlgebra( (SA1+r12*sqrt(SA1*SA2))*solve(SA), name="g1"),

mxAlgebra( (SA2+r12*sqrt(SA1*SA2))*solve(SA), name="g2"),

# AC covariances

mxAlgebra(g1*(CAC), name="CA1"),

mxAlgebra(g2*(CAC), name="CA2"),

#

# constraint positive definite covariance A1,C and A2,C matrices

#

mxAlgebra(rbind(cbind(SA1,CA1),cbind(CA1,SC)), name='SAC_1'),

mxAlgebra(rbind(cbind(SA2,CA2),cbind(CA2,SC)), name='SAC_2'),

mxAlgebra(eigenval(SAC_1), name='eval_1'),

mxAlgebra(eigenval(SAC_2), name='eval_2'),

# if you do not want the pos def constraint comment the following out using #

mxConstraint(eval_1 > z21, name='evalc_1'),

mxConstraint(eval_2 > z21, name='evalc_2'),

#

# MZ latent covariance (E A1 A2 C E A1 A2 C)

#

mxAlgebra(expression=rbind(

# E A1 A2 C E A1 A2 C

cbind(SE, 0, 0, 0, 0, 0, 0, 0), # E

cbind( 0, SA1, C12, CA1, 0, SA1, C12, CA1), # A1

cbind( 0, C12, SA2, CA2, 0, C12, SA2, CA2), # A2

cbind( 0, CA1, CA2, SC, 0, CA1, CA2, SC), # C

#

cbind( 0, 0, 0, 0, SE, 0, 0, 0), # E

cbind( 0, SA1, C12, CA1, 0, SA1, C12, CA1), # A1

cbind( 0, C12, SA2, CA2, 0, C12, SA2, CA2), # A2

cbind( 0, CA1, CA2, SC, 0, CA1, CA2, SC)),name='latSmz'), # C

#

# DZ latent covariance (E A1 A2 C E A1 A2 C)

#

mxAlgebra(expression=rbind(

cbind(SE, 0, 0, 0, 0, 0, 0, 0),

cbind( 0, SA1, C12, CA1, 0, .5*SA1, .50*C12, CA1),

cbind( 0, C12, SA2, CA2, 0, .50*C12, .50*SA2, CA2),

cbind( 0, CA1, CA2, SC, 0, CA1, CA2, SC),

#

cbind( 0, 0, 0, 0, SE, 0, 0, 0),

cbind( 0, .50*SA1, .50*C12, CA1, 0, SA1, C12, CA1),

cbind( 0, .50*C12, .50*SA2, CA2, 0, C12, SA2, CA2),

cbind( 0, CA1, CA2, SC, 0, CA1, CA2, SC) ),name='latSdz'),

#

# MZ and DZ covariance matrices

mxAlgebra(expression=(LY%*%latSmz%*%t(LY))[1:3,1:3],name="SMz"),

mxAlgebra(expression=LY%*%latSdz%*%t(LY),name="SDz")

#

)

#

# ---------------- end PARS

# MZ model

mzModel <- mxModel(name = "MZ",

mxCI(c('cAC')),

mxMatrix(type="Full", nrow=1, ncol=3, free=TRUE, values= c(8,0,8), label=c("mT","mp","mT"), name="expMeanMz"),

# Algebra for expected variance/covariance matrix in MZ

mxAlgebra(expression=(PARS.LY%*%PARS.latSmz%*%t(PARS.LY))[1:3,1:3],name="expCovMz"),

# data

mxData(observed=dmz, type="raw"),

mxExpectationNormal(covariance="expCovMz", means = "expMeanMz", vnames[1:3]),

mxFitFunctionML()

)

#

dzModel <- mxModel(name = "DZ",

mxCI(c('cAC')),

mxMatrix(type="Full", nrow=1, ncol=4, free=TRUE, values= c(8,0,8,0), label=c("mT","mp","mT","mp"), name="expMeanDz"),

# Algebra for expected variance/covariance matrix in DZ

mxAlgebra(expression=PARS.LY%*%PARS.latSdz%*%t(PARS.LY),name="expCovDz"),

mxData(observed=ddz, type="raw"),

mxExpectationNormal(covariance="expCovDz", means = "expMeanDz", vnames[1:4]),

mxFitFunctionML()

)

# fit the model

ACModel <- mxModel(model="TwAC", mzModel, dzModel, part1,

mxFitFunctionMultigroup( c("MZ","DZ") ))

#

#Run MX

#ACModelFit <- mxRun(ACModel, intervals=T)

# better use use extra tries

ACModelFit <- mxTryHard(ACModel, extraTries = 200)

#

# drop the parameter cAC

#

ACModel2=omxSetParameters(ACModel,label=c('cAC'), free=c(F), value=0)

#ACModel2Fit <- mxRun(ACModel2)

ACModel2Fit <- mxTryHard(ACModel2, extraTries = 200)

test1=mxCompare(ACModelFit, ACModel2Fit)

# parameter estimates and test of AC covariance

summary(ACModelFit)

print(test1)

# ----------------------------------------------- end

**Results obtained with the OpenMx code as given above.**

1) Parameters estimates (with annotation)

> summary(ACModelFit)$parameters[,c(1,5)]

name Estimate *annotation*

1 mT 8.000000e+00 *(mean of phenotype)*

2 mp -9.203188e-08 *(mean of PRS)*

3 p 9.999998e-01 *(p scaling of PRS)*

4 sA1 1.998000e-01 *(σ_Ap_^2^)*

5 sA2 2.997009e-01 *(σ_Aq_^2^)*

6 sC 2.996988e-01 *(σ_C_^2^)*

7 sE 1.997993e-01 *(σ_E_^2^)*

8 cAC 1.160729e-01 *(σ_AC_)*

2) LogLikelihood ratio test of *σ_AC_*=0. The test statistic equals 13.0264

> print(test1)

base comparison ep minus2LL df AIC diffLL diffdf p

1 TwAC <NA> 8 12993.54 6992 -990.4557 NA NA NA

2 TwAC TwAC 7 13006.57 6993 -979.4293 13.0264 1 0.0003071305
